# Three-Dimensional Assessment of Dental Enamel Microcrack Progression After Orthodontic Bracket Debonding Using Optical Coherence Tomography

**DOI:** 10.3390/jfb16010007

**Published:** 2024-12-30

**Authors:** Ahmed Haj Hamdan, Sm Abu Saleah, Daewoon Seong, Naresh Kumar Ravichandran, Ruchire Eranga Wijesinghe, Sangyeob Han, Jeehyun Kim, Mansik Jeon, Hyo-Sang Park

**Affiliations:** 1 Department of Orthodontics, School of Dentistry, Kyungpook National University, Daegu 41940, Republic of Korea; drahmadhamdan7@knu.ac.kr; 2 ICT Convergence Research Center, Kyungpook National University, Daegu 41566, Republic of Korea; abu.saleah@knu.ac.kr; 3 School of Electronic and Electrical Engineering, College of IT Engineering, Kyungpook National University, 80, Daehak-ro, Buk-gu, Daegu 41566, Republic of Korea; smc7095@knu.ac.kr (D.S.); syhan850224@knu.ac.kr (S.H.); jeehk@knu.ac.kr (J.K.); 4 Center for Scientific Instrumentation, Korea Basic Science Institute, 169-148 Gwahak-ro, Yuseong-gu, Daejeon 34133, Republic of Korea; nareshr.9169@gmail.com; 5 Department of Electrical and Electronic Engineering, Faculty of Engineering, Sri Lanka Institute of Information Technology, Malabe 10115, Sri Lanka; eranga.w@sliit.lk; 6 Center for Excellence in Informatics, Electronics & Transmission (CIET), Sri Lanka Institute of Information Technology, Malabe 10115, Sri Lanka

**Keywords:** enamel, microcracks, metal brackets, ceramic brackets, orthodontic brackets, optical coherence tomography (OCT), 3D imaging

## Abstract

The current study aimed to quantify the length progression of enamel microcracks (EMCs) after debonding metal and ceramic brackets, implementing OCT as a diagnostic tool. The secondary objectives included a three-dimensional assessment of EMC width and depth and the formation of new EMCs. OCT imaging was performed on 16 extracted human premolars before bonding and after debonding. Debonding was conducted with a universal Instron machine, with ARI values recorded. Additionally, 2D and 3D OCT images were employed to detect EMC formation and progression. Enface images quantified the length, width, and number of EMCs, and the length and width were analyzed using Image J (1.54f) and MATLAB (R2014b), respectively. Sagittal cross-sectional images were used for EMC depth analysis. A paired *t*-test showed significant differences in the length, width, and number of EMCs after debonding (*p*-value < 0.05), while the Wilcoxon non-parametric test indicated significant EMC depth changes (*p*-value < 0.05). No significant results were identified for the EMC number in ceramic brackets and EMC depth in metal brackets. Three-dimensional OCT imaging monitored existing EMCs at higher risk of progression and detected new EMCs following orthodontic bracket debonding. This study provides novel insights into EMC progression regarding the length, width, depth, and number after debonding.

## 1. Introduction

Enamel microcracks (EMCs) are characterized by irreversible damage to the enamel surface and are a common iatrogenic side effect of orthodontic debonding using metal or ceramic brackets [1,2,3]. EMCs often lead to the formation of unesthetic stains and facilitate plaque accumulation, thus increasing the enamel surface’s susceptibility to caries development and demineralization [4]. Although the prevalence of EMCs is similar between patients with and without orthodontic treatment, these microcracks tend to be more noticeable after bracket debonding, highlighting the importance of early detection and monitoring [5].

Previous studies have employed a variety of assessment tools, including visual inspection, transillumination, stereomicroscopy, scanning electron microscopy (SEM), quantitative light-induced fluorescence (QLF), periapical X-rays, and micro-computed tomography (µCT), to evaluate EMCs on tooth surfaces. However, these methods have several limitations, including an inability to detect deeper injuries [6], failure to capture EMCs when the light beam is parallel to its direction [7], high costs associated with complex imaging processes [8,9], and increased risk associated with radiation exposure [10,11]. These limitations have induced the demand for a safe and reliable diagnostic tool for the assessment of EMCs.

Optical coherence tomography (OCT) has emerged as a key advancement in the fields of medicine and dentistry due to its ability to provide high-resolution, high-contrast, real-time imaging in a non-invasive and non-destructive manner [12]. It has been widely adopted in the field of ophthalmology [13,14] and has also been utilized in dentistry [15] to monitor the progression of dental caries [16,17,18,19,20], evaluate white spot lesions [21,22], appraise dental restorations [23,24], characterize soft tissues [25,26,27], and determine biofilm thickness [28,29]. Additionally, OCT has also been used in the field of orthodontics [30] to examine the enamel layer [31], assess the presence of adhesive remnants [32], evaluate the bracket–tooth interface [33], quantify demineralization [34], explore biofilm retention on the surface of orthodontic brackets [35], and characterize cortical bone following micro-implant insertion [36]. Recently, the OCT system has been introduced in EMC detection [37,38,39,40]. However, limited research has employed OCT to diagnose EMCs as an iatrogenic side effect of bracket debonding.

The damage of bracket debonding is not only related to EMC formation but also to EMC propagation. As obvious EMCs are easily identified and produce more extensive consequences, determining the progression of pre-existing EMCs may be more imperative than assessing that of new EMCs. The formation of new EMCs has been well documented [41]; however, there is inadequate research evaluating the propagation of EMCs despite their importance.

Therefore, this study primarily aims to quantify the length progression of EMCs following the debonding of metal and ceramic brackets, using OCT as a diagnostic tool. The secondary objectives of this study are to conduct a three-dimensional assessment of EMCs in terms of width and depth and to evaluate the formation of new EMCs.

## 2. Materials and Methods

### 2.1. Sample Selection

This study was approved by the Institutional Review Board of Kyungpook National University Dental Hospital (No. KNUDH-2022-11-02-00). A total of 22 premolars, which were extracted for orthodontic treatment, were collected and used within six months of extraction. All teeth were assessed to ensure a sound enamel surface for the bonding of metal and ceramic brackets. Samples with a history of orthodontic, endodontic, or restorative treatment and those with fractures, caries, or damage were excluded. First, a preliminary study was performed on four randomly chosen samples to assist the research design, including a positive control group wherein debonding was conducted under massive force to ensure the progression of EMCs. Then, the minimum sample size required to detect the difference in means of a two-tailed dependent *t*-test with a significance level of 0.05 was calculated using G-Power, based on an effect size of 1.65, obtained from previous evidence on the EMC length [42,43]. The findings showed that a minimum sample size of seven specimens is required to achieve a power of 95% for each bracket type. Therefore, the total sample size for all brackets was 14 samples. An additional four samples were included to account for any attrition during this experiment; thus, the final sample size was 18 teeth.

### 2.2. Sample Preparation

This study was carried out following the Declaration of Helsinki. All remnants were removed from the tooth surface using a scaler. The samples were stored in 0.9% saline solution (Krinzo; JW Group, Seocho-gu, Seoul, Republic of Korea) in a glass container to prevent chemical interactions between the samples and the container after each step. Moreover, a total of 18 cube-shaped, chemically cured acrylic resin blocks measuring 20 mm in length, width, and depth were prepared using a plastic mold. The roots of the premolars were embedded in the blocks, and the buccal surfaces were mounted to ensure a flattened surface. The acrylic resin blocks provided a flat surface, minimizing the need to tilt the samples during imaging and ensuring adequate light distribution during OCT scanning. They also provided a stable grip for the Instron machine during debonding. The buccal surfaces of the samples were kept free from contamination.

### 2.3. OCT Imaging System

**Figure 1:**
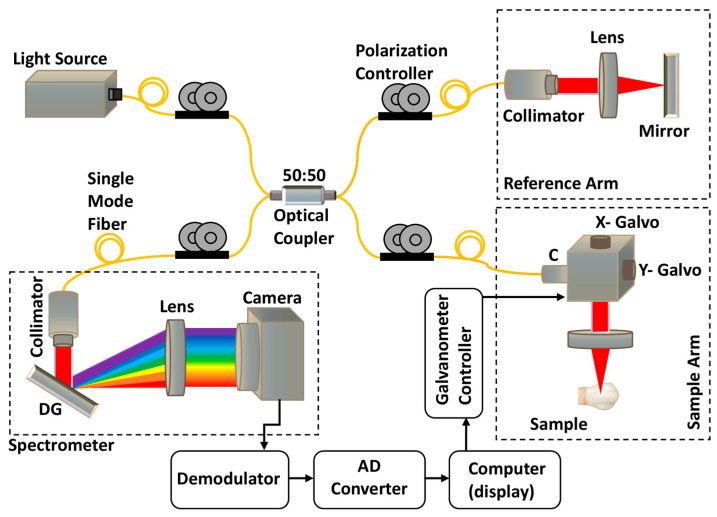
Schematic diagram of optical coherence tomography system.

The spectral-domain optical coherence tomography (SD-OCT) system used in this study included a broadband light source (EXS210045-01) with a central wavelength of 1310 nm and a full-width at half-maximum (FWHM) bandwidth of 100 nm (Figure 1). The light source had an output power of 10 mW, with the beam split 50:50 in the fiber-based Michelson interferometer before being fed to both the reference and sample arms. The output power of the reference arm was kept constant throughout the study duration, while that of the sample arm had a mean value of 4.2 mW. Two Galvo scanners (GVS002, Thorlabs, Newton, NJ, USA) and a 75 mm focusing lens (AC508-075-C, Thorlabs, USA) were attached at the end of the sample arm to scan an area measuring 13 × 16 mm on the buccal aspect of the teeth. The lights reflected from the reference arm mirror and the sample were interfered with at the fiber coupler before being delivered to the spectrometer, utilizing a 2048-pixel line scan camera (GL2048L-10A-ENC-STD-210). This system had axial and lateral resolutions of 7.55 and 17 µm, respectively, in air, with a frame rate of 50 frames per second. Therefore, the 1000 B-scan images acquired in 20 s were used to form 3D (three-dimensional) OCT images. The length, width, depth, and number of the EMCs were analyzed using OCT images taken from the 3D images of the samples.

### 2.4. Baseline Measurement (Before Bonding)

Three-dimensional OCT imaging of the entire buccal surfaces of all samples was carried out prior to the treatment process (intact enamel surface) and used as the control group, referred to as “before bonding” in this study. Each sample was returned to the saline solution after scanning. The longest EMC was identified and selected to be monitored for any progression throughout the experiment.

### 2.5. Bonding Procedure

All samples were randomized into two groups (n = 9 each), receiving either metal (AbsoClusion; Dentos Inc., Dalseo-Gu, Daegu, Republic of Korea) or ceramic (Zeemas C; Dentos Inc., Dalseo-Gu, Daegu, Republic of Korea) brackets. The buccal surfaces of the samples were polished with a fluoride-free pumice, etched for 30 s with 37% phosphoric acid (ETCH-37™ w/BAC, BISCO, Inc., Schaumburg, IL, USA), and then rinsed with water for 10 s using a three-in-one syringe. The teeth were air-dried for 10 s with an oil-free air source, followed by the application of a primer (TransbondTM XT, 3M Unitek, Monrovia, CA, USA). The brackets were bonded using a standardized amount of adhesive (Transbond™ XT Light Cure Adhesive; 3M Unitek, Monrovia, CA, USA), measured with a precision electronic scale (0.1 mg sensitivity; Mettler-Toledo International Inc., Greifensee, Switzerland). Furthermore, 400 g of pressure was applied with a dynamometer (Tension Gauge DTN-50, TECLOCK Co., Ltd., Nagano, Japan) for bonding. Each bracket was positioned at a height of 4 mm along the long axis of the premolar using a bracket gauge (Bracket Placement Marker/Measuring Gauge; Ormco Corporation, Orange, CA, USA) [44]. After removing excess cement, the samples were cured using a high-power light-emitting diode curing unit with an intensity of 3000 mW/cm^2^ ± 10% (Noblesse Light-Curing Unit; Max Dental Co., Gyeonggi-do, Seoul, Republic of Korea). The light-curing was implemented for 5 s from the mesial, distal, cervical, and occlusal directions with a 5 mm distance. The samples were then stored in distilled water (sterile distilled water; JW Group, Seocho-gu, Seoul, Republic of Korea) at room temperature for 24 h before undergoing debonding (carried out by one operator—A.H.H) [45].

### 2.6. Debonding Procedure and Adhesive Removal

**Figure 2:**
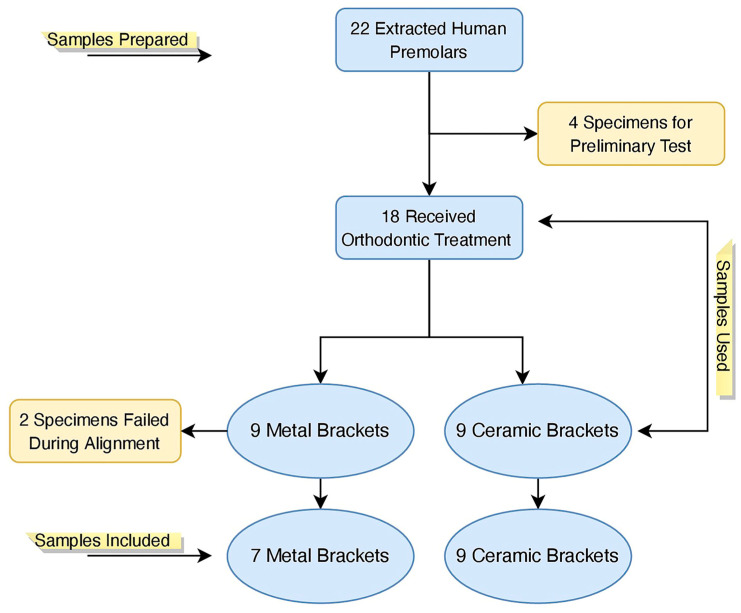
Study flow diagram.

The specimens were secured in a jig attached to the base plate of the Instron machine (Instron, Illinois Tool Works Inc., Norwood, MA, USA), and a chisel-edge plunger was positioned perpendicular to the bonded bracket–tooth interface. The cross-head speed was set to 0.5 mm/min, and a cell load of 1 kN was applied to each bracket for debonding [46]. Two specimens failed during sample alignment; thus, the final sample size in the current study was 16 teeth (Figure 2). The shear bond strength (SBS), reported in MegaPascals (MPa), was calculated by dividing the force applied by the bracket area (metal: 3.4 × 3.4 mm; ceramic: 3.4 × 3.0 mm).

The residual resin was first marked with a pencil by one operator (A.H.H) under 2.5× loupe magnification power and then removed using a low-speed, contra-angle handpiece with a suitable carbide bur (RA-6 No. 310006, SS White Dental, Lakewood, NJ, USA) at a speed of 30,000 rpm with water cooling. The adhesive remnants index (ARI) was defined as follows: Score 0—no composite resin left on the tooth’s surface; score 1—less than half of the composite resin left on the tooth’s surface; score 2—more than half of the composite resin left on the tooth’s surface; and score 3—all of the composite resin left on the tooth’s surface [47].

### 2.7. After Debonding Measurement (After Debonding)

**Figure 3:**
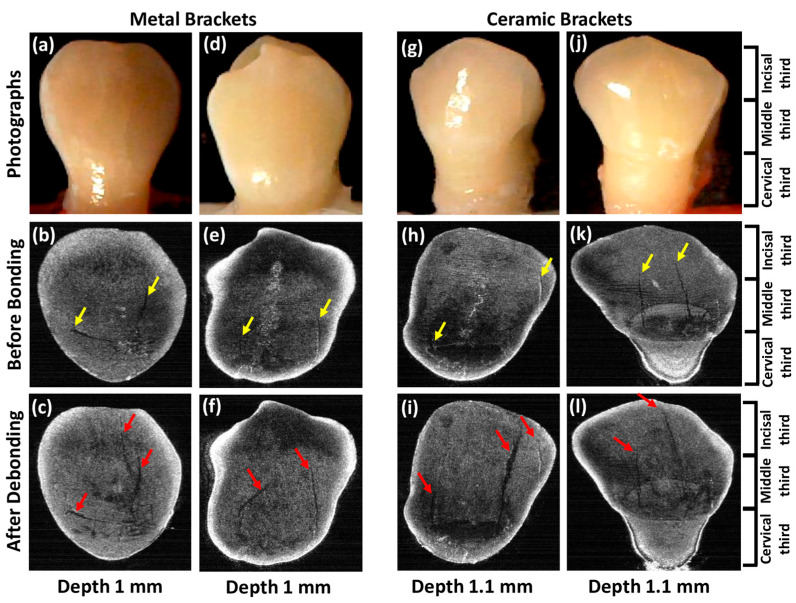
Enface OCT images showing enamel crack progression before bonding and after debonding by group. The before-bonding dental conditions of dental samples are presented in the photographs (**a**,**d**,**g**,**j**). The yellow arrows show existing cracks before bonding (**b**,**e**,**h**,**k**), while the red arrows show the visible progression of the cracks after debonding (**c**,**f**,**i**,**l**). The enface images were taken at a depth of 1 mm from the buccal surface in the metal group and 1.1 mm from the buccal surface in the ceramic group.

The experimental groups consisted of all OCT records after debonding and adhesive removal, while the OCT scans were performed in a blinded manner by one operator S.A.S. A total of 1000 two-dimensional (2D) OCT images of each sample were acquired before and after debonding to assess the progression and development of the EMCs as a result of bracket debonding. The OCT scan allowed testing of pre-treatment and post-treatment conditions at different regions of interest (ROIs), such as cervical, middle, and incisal thirds (as shown in Figure 3).

### 2.8. Data Analysis

Each sample scan consisted of 1000 2D images, which were scanned before bonding and after debonding from the buccal side. The obtained 2D images were used to reconstruct the 3D OCT image of the entire buccal surface using Amira (Amira software, Version 5.3.3, Zuse Institute Berlin; Thermo Fisher Scientific, Waltham, MA, USA) software. The uniformity of the before and after scans was challenging; however, this was overcome with the use of acrylic resin blocks, allowing the scan to be obtained from the same angle and distance at all times. EMC length, width, and quantity measurements were performed using the enface image obtained from the entire reconstructed buccal surface of the sample (as shown in Figure 3). The image was taken at a depth of 1 or 1.1 mm from the outer buccal surface of the samples, depending on the enface image quality of the individual sample. Each sample contained one or more EMCs. All EMC lengths of each sample were measured, and the longest EMC was traced to measure its width and depth since the longest EMC could have greater iatrogenic effects [4].

In this study, all the treatment steps were performed carefully to avoid enamel damage. The enface images were used for each sample to assess the EMC length before the treatment, which was considered as the control group. This protocol allowed the identification of the EMCs before bonding and after debonding conditions by targeting the same enface image for each sample. The length was measured using ImageJ (ImageJ software, version 1.54f, U.S. National Institutes of Health, Bethesda, MD, USA). The initial and final measurement points were taken from the enface images, where both points can be clearly seen (as shown in Figure 3). The segmented line function of the ImageJ software was used to accommodate the cracks that were not perfectly straight. The length of the curve crack was measured by drawing the segmented line next to the crack. 

**Figure 4:**
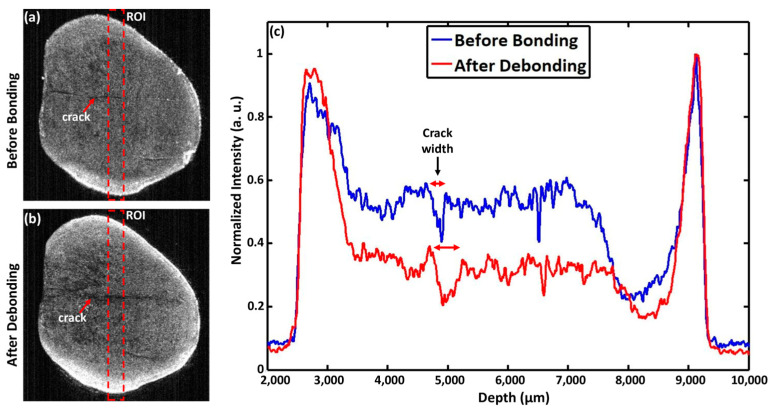
Measurement of EMC width progression (**a**) before bonding and (**b**) after debonding using 2D enface images. The red dotted boxes indicate the region of interest (ROI) for obtaining depth-intensity profiles, while the red arrows indicate the enamel cracks. (**c**) Depth intensity profiles derived from the enface images of the samples before bonding and after debonding.

Width data were analyzed with the aid of MATLAB (MATLAB software, Version R2014b, the MathWorks, Natick, MA, USA) by developing and adopting an algorithm for the OCT image intensity analysis (A-scan analysis) at ROIs of the same enface images, taken at a depth of 1 or 1.1 mm from the outer buccal surface of the samples. The same depth was maintained for comparing the width of EMCs before bonding and after the debonding. The first enface image from the top of the buccal surface was considered as the reference for obtaining the target enface image in the desired depth. ROIs were chosen from the enface images of both groups, where the width of the EMCs was visualized for comparison through A-scan profiles (as marked by the double arrows in Figure 4). The width was measured from a single point/area (as indicated by the red dotted box in Figure 4) to compare the width of the EMCs before and after bracket debonding. The sagittal 2D cross-section was used for the EMC depth assessment and an index scale defined by Imai et al. [39] was applied as follows: 1—no crack, intact surface; 2—superficial enamel crack extending <50% of the enamel thickness; 3—deep enamel crack extending >50% of the enamel thickness but not extending up to the dentin–enamel junction (DEJ); 4—whole-thickness enamel crack extending up to the DEJ; and 5—dentin crack extending beyond the DEJ.

### 2.9. Statistical Analysis

The Shapiro–Wilk test was first used to confirm the normal distribution of the data. A paired *t*-test was employed to compare the length, width, and number of EMCs before and after debonding, while the Wilcoxon signed-rank test was used to compare the depth of EMCs. Pearson’s and Spearman’s correlation analyses were implemented to explore the associations between the variables. All statistical analyses were performed using IBM SPSS (version 26.0; SPSS Inc., Chicago, IL, USA), and the level of statistical significance was set at a *p*-value of <0.05.

## 3. Results

No statistically significant differences in EMC length, width, depth, and number were observed among the samples at baseline.

### 3.1. EMC Length

The mean EMC length before bonding was 4.41 ± 1.76 mm in the total sample, which was increased to 5.32 ± 1.60 mm (*p*-value < 0.001). There was an increase in EMC length in most samples, except for the long pre-existing EMC lengths showing no progression (Supplement Data Table S1). The mean EMC lengths of the metal and ceramic brackets at baseline were 4.54 ± 1.73 mm and 4.31 ± 1.89 mm, which increased to 5.14 ± 1.43 mm and 5.47 ± 1.80 mm after debonding, respectively (metal: *p*-value = 0.033; and ceramic: *p*-value = 0.002; Table 1 and Figure 3).

### 3.2. EMC Width

The mean width of the EMCs before bonding was 175.50 ± 56.76 µm in the total sample, which increased significantly to 359.93 ± 110.38 µm (*p* < 0.001). All samples showed an increase in EMC width after debonding (Supplement Data Table S1). The mean EMC width increased from 185.71 ± 54.49 µm to 364.00 ± 128.47 µm in the metal brackets (*p*-value = 0.004) and from 167.55 ± 60.43 µm to 356.77 ± 102.17 µm in the ceramic brackets after debonding (ceramic, *p*-value = 0.002; Table 1 and Figure 4).

### 3.3. EMC Depth Index

**Figure 5:**
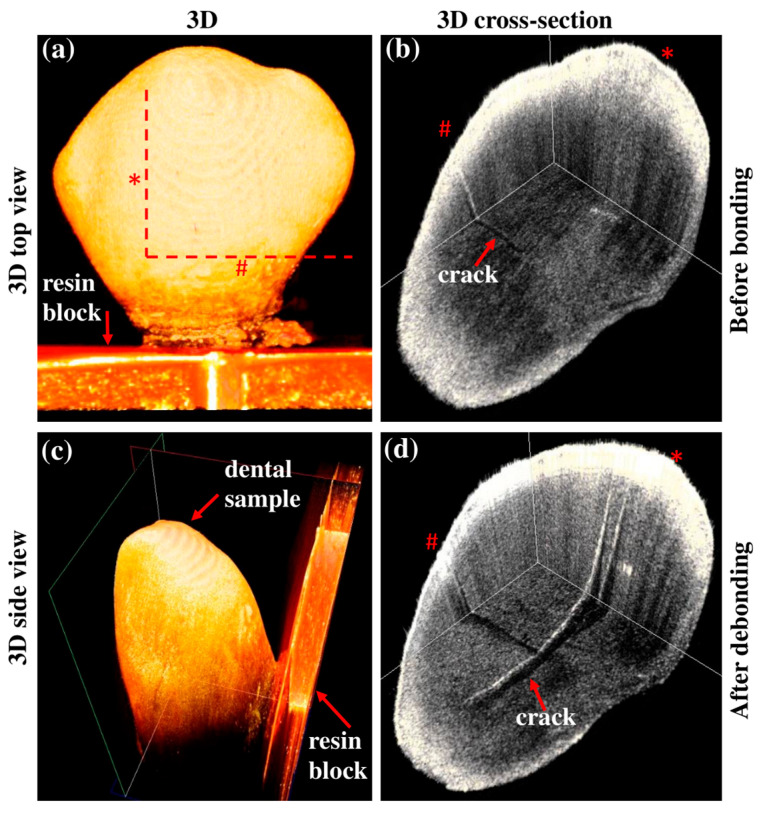
Three-dimensional cross-sectional images illustrating EMC progression: (**a**) no cracks visible on the buccal surface before bonding; (**b**) pre-existing crack with a depth index score of 3 seen on the enamel surface before bonding (red arrow); (**c**) side view of the dental 3D image; (**d**) formation of new EMC and (*) vertical and (#) horizontal expansion of pre-existing crack (increase in depth index score from 3 to 5) visible after debonding (red arrows). The red dotted lines in (**a**) indicate the 3D cross-sectional positions of (**b**,**d**).

**Figure 6:**
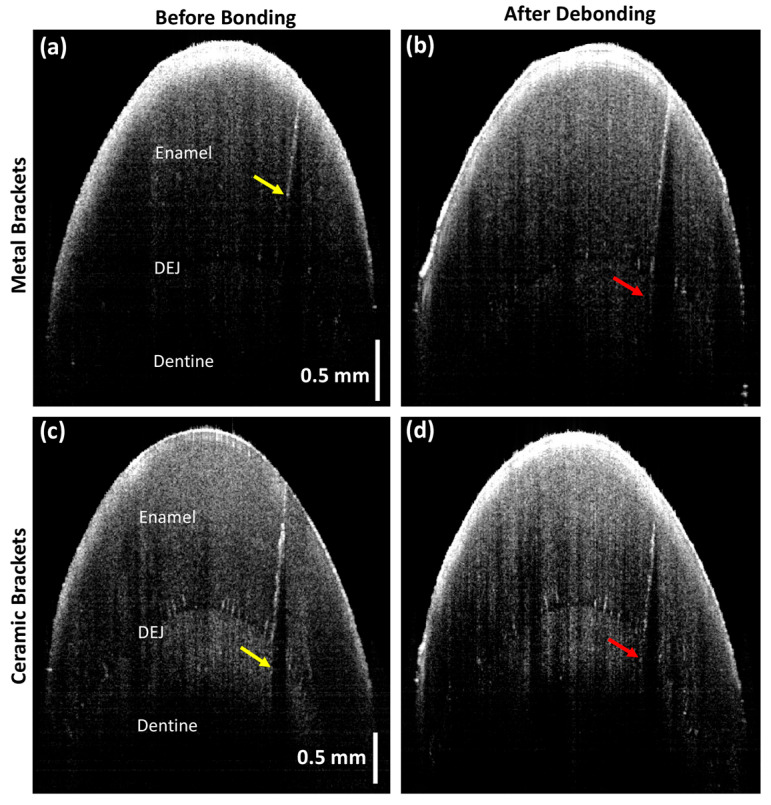
Two-dimensional sagittal cross-sectional OCT images indicating EMC progression. The cross-sectional view shows the enamel layer, dentin–enamel junction (DEJ), and dentine layer. (**a**,**c**) EMC depth index before bonding (yellow arrows) and (**b**,**d**) EMC depth index progression after debonding (red arrows) in the metal and ceramic brackets, respectively.

Progression in the depth of the EMCs was observed in three samples with metal brackets and five with ceramic brackets. The differences were statistically significant in the total sample (*p*-value = 0.009) and the ceramic group (*p*-value = 0.038) but not in the metal group (Table 1, Figure 5 and Figure 6).

### 3.4. EMC Number

The mean number of EMCs before bonding was 1.93 ± 0.57 in the total sample and increased after debonding to 2.37 ± 0.61 (*p*-value = 0.004). The EMCs number of metal and ceramic brackets were 2 ± 0.57 and 1.88 ± 0.60, respectively. These numbers were increased after debonding in metal brackets to 2.57 ± 0.53 (*p*-value = 0.030) and 2.22 ± 0.66 in the ceramic brackets (Table 1, Figure 3).

### 3.5. Shear Bond Strength (SBS) and Adhesive Remnant Index (ARI)

The mean SBS value was 16.11 ± 5.29 MPa in the total sample, 15.12 ± 5.91 MPa in the metal group, and 16.88 ± 4.98 MPa in the ceramic group. No statistically significant differences in SBS were observed between the brackets (Table 2). A total of six samples exhibited an ARI score of 1, and only one sample scored 3 in the metal group, while five samples in the ceramic group scored 1, two samples scored 2, and two samples scored 3. Therefore, the ceramic brackets exhibited greater resin remnants on the tooth surface, although this difference was not significant (Table 3). The correlation between the variables was then tested; however, Pearson’s and Spearman’s correlation tests did not indicate any associations between the EMC length, width, depth, and number and the SBS or ARI (Table 4).

## 4. Discussion

Although previous studies have extensively examined the formation of new EMCs after debonding, few have explored EMC progression [41]. The current study was the first to carry out a 3D examination of EMC progression after bracket debonding and provide novel insights into the behavior of EMCs located closer to the DEJ (Figure 5). The OCT system facilitated non-destructive, non-invasive, and accurate enamel sectioning without the risks of radiation exposure. Previous studies primarily examined the progression of EMCs located on the outer buccal surface, using 2D images to categorize them based on location or direction [8,48]. In contrast, the current study used OCT to identify the propagation of cracks in terms of elongation, expansion, or deepening based on the size and orientation of the EMCs. This progression of EMCs may relate to the enamel prismatic and inter-prismatic crystalline structures [49,50].

The progression and formation of EMCs are dependent on the composition of the enamel at the site of debonding force application. Dental enamel contains minimal organic components such as enamel tufts, which are hypocalcified defective sites that weaken the inter-rod structure and facilitate the transmission of shear forces during debonding, leading to the formation of cracks [50]. This is typically observed when the debonding stresses occur at the adhesive–tooth interface [5].

This study found a significant propagation in EMCs after debonding, with an overall average length increase of approximately 20.6% (metal: 13.21%; ceramic: 26.9%). The EMC width had increased significantly by 105% (metal: 96%; ceramic: 112%). Since EMC depth measurements can be precisely achieved for EMCs extended only within the enamel layer, the EMC depth index scale was implemented in the current study. The depth index showed that 50% of the total sample displayed depth progression (metal: 42.8%; ceramic: 55.5%). In addition, the 2D and 3D OCT images confirmed the formation of new EMCs after debonding (Figure 3 and Figure 5). Approximately 43.8% of the study sample (n = 7/16) exhibited newly formed EMCs (metal: 66.6%; ceramic: 33.3%). However, the progression of EMCs was not coincident with the recent systematic review and meta-analysis study [41], which may be attributed to the advanced imaging with OCT. The wide range in the standard deviation in the current study was related to the variation in the EMC size between the samples but not to the measurements. However, the statistical tool, a paired *t*-test, allowed the detection of the progression of EMCs. Nevertheless, the current study introduced a novel approach by incorporating OCT enface images and the ImageJ software to measure the EMC length (Figure 3) and the depth-intensity profile algorithms that provided micrometer measurements of the EMC width (Figure 4).

Interestingly, the EMC width has the highest propagation percentage when compared to the EMC length, depth, and number. While the progression of the EMC length and number requires the breakage of intact enamel rods, the enlargement of the EMC width occurs at the previously broken rods, resulting in greater damage. On the other hand, the propagation of the EMC depth requires the fracture of the parallel and perpendicular-oriented rods that resist the formation of EMCs and their progression [51].

The existing literature suggests that visible EMCs vary in size (typically, a length of 2.67–12.42 mm; width, 1.81–13.04 µm) [41], which significantly increases the likelihood of plaque accumulation, staining, sensitivity, demineralization, or caries development [4]. According to Kalloniatis and Luu, an individual would be able to see objects of 6–29 microns, depending on their age [52]. The advantage of OCT is that it allows the measurement of EMCs on a micrometer scale. The present study found that debonding leads to an increase in crack size (20.6% in length; 105% in width), indicating that existing EMCs are at a higher risk of progression upon debonding, leading to more adverse effects, regardless of the bracket material. Thus, using OCT prior to the commencement of orthodontic treatment can help patients visualize existing EMCs before treatment. Therefore, the inclusion of EMC progression in the informed consent is crucial, especially in adults who have pre-existing cracks [53]. In addition, OCT could guide clinicians in the decision-making process, particularly when considering the type and design of appliances. Although the current results demonstrated EMC progression, it does not mean that the use of metal or ceramic brackets is not recommended. Rather, a risk/benefit analysis should be meticulously performed. Moreover, aligner orthodontic therapy could serve as an alternative if it could be executed without the need for bonding and debonding [54,55]. Furthermore, debonding of fixed orthodontic appliances can be modified to reduce stress on the enamel surface by holding the bracket gingival wing at a 45° angle using a bracket-removing plier and by applying peeling-type forces toward the bracket–adhesive interface [56,57] (pp. 787–791). Alternative measures include the use of lasers to remove metal and ceramic brackets [58] and slow-speed handpieces and carbide burs to remove adhesives without generating harmful forces on the enamel surface [59].

The current study achieved its main aim of evaluating EMC length progression after bracket debonding as well as its width, depth, and number. The in vitro study design simplified the quantification of EMC characteristics using OCT imaging. However, discrete EMC depth measurements were not possible due to the extension of the EMCs, exceeding the OCT capturing capacity beyond the DEJ. This particular limitation can be overcome with the integration of nano/micro-CT imaging in future studies. A second limitation of the current study was that the OCT images of the cervical area of the teeth had poor resolution due to the inclination of the tooth surface, which hindered the accurate evaluation of cervical EMCs. If the EMC extended on the whole buccal surface before bonding, the progression can only be detected in the width direction, not in length, after debonding. Another limitation was that the dimensions of the EMCs may have been slightly exaggerated due to tooth desiccation. Moreover, bonding strength under in vitro conditions is typically higher than those observed clinically due to better moisture control and the absence of saliva. The application of OCT in clinical and in vivo settings appears promising. In the future, OCT with an extended depth of focus could detect cracks that extend beyond the DEJ, facilitating an improved depth assessment. Finally, the comparison between metal and ceramic was out of the scope of the current study; therefore, further studies using a larger sample size are necessary.

## 5. Conclusions

In conclusion, the current study used OCT as a diagnostic tool to quantify the length, width, depth, and number of EMCs and examine their progression following orthodontic bracket debonding. OCT enabled 2D and 3D visualization and monitoring of EMCs closer to the DEJ, as existing EMCs are considered to be at a higher risk of progression during bracket bonding and debonding. In addition, the advanced OCT technique was utilized in conjunction with ImageJ software and depth-intensity profile algorithm tools to measure an EMC’s length and width. The results confirmed that the iatrogenic side effect of the bracket debonding is not limited to EMC formation but also to the EMC propagation in terms of length, width, and depth. The definitive conclusions regarding variations in risk by orthodontic bracket type or bracket repositioning could be drawn by future studies.

## Figures and Tables

**Table 1:** Comparison of the length, width, depth, and number of enamel microcracks before bonding and after debonding.

		Before (Mean ± SD)	After (Mean ± SD)	*p*-Value
Length (mm) ^a^				
	Metal (n = 7)	4.54 ± 1.73	5.14 ± 1.43	0.033 *
	Ceramic (n = 9)	4.31 ± 1.89	5.47 ± 1.80	0.002 **
	Total (n = 16)	4.41 ± 1.76	5.32 ± 1.60	<0.001 ***
Width (μm) ^a^				
	Metal (n = 7)	185.71 ± 54.49	364.00 ± 128.47	0.004 **
	Ceramic (n = 9)	167.55 ± 60.43	356.77 ± 102.17	0.002 **
	Total (n = 16)	175.50 ± 56.76	359.93 ± 110.38	<0.001 ***
Depth Index ^b^				
	Metal (n = 7)	4.00 ± 1.15	4.57 ± 0.78	0.102
	Ceramic (n = 9)	3.11 ± 1.05	3.88 ± 1.26	0.038 *
	Total (n = 16)	3.50 ± 1.15	4.19 ± 1.10	0.009 **
Number ^a^				
	Metal (n = 7)	2 ± 0.57	2.57 ± 0.53	0.030 *
	Ceramic (n = 9)	1.88 ± 0.60	2.22 ± 0.66	0.081
	Total (n = 16)	1.93 ± 0.57	2.37 ± 0.61	0.004 **

* *p* < 0.05; ** *p* < 0.01; *** *p* < 0.001 (^a^ Paired *t*-test; ^b^ Wilcoxon signed-rank test).

**Table 2:** Mean shear bond strength by type of bracket used.

Group	N	Mean ± SD	Minimum	Maximum
Metal	7	15.12 ± 5.91	4.17	20.36
Ceramic	9	16.88 ± 4.98	9.17	24.61
Total	16	16.11 ± 5.29	4.17	24.61

Shear bond strength (SBS) MPa. No statistically significant differences were observed between the groups (independent *t*-test *p*-value = 0.529).

**Table 3:** Distribution of adhesive remnant index (ARI) scores.

Group	0	1	2	3
Metal (n = 7)	0 (0%)	6 (85.7%)	0 (0%)	1 (14.3%)
Ceramic (n = 9)	0 (0%)	5 (55.6%)	2 (22.2%)	2 (22.2%)
Total (n = 16)	0 (0%)	11 (68.7%)	2 (12.6%)	3 (18.7%)

ARI score 0—no composite resin left on the tooth’s surface; score 1—less than half of the composite resin left on the tooth’s surface; score 2—more than half of the composite resin left on the tooth’s surface; and score 3—all of the composite resin left on the tooth’s surface. No statistically significant differences were observed between the groups (independent Wilcoxon rank-sum test, *p*-value = 0.271).

**Table 4:** Correlation between SBS and ARI scores and enamel microcrack length, width, depth, and number.

			Length	Width	Depth Index	Number
Total (n = 16)	SBS ^a^	rho	0.373	0.013	0.114	0.101
		*p*-value	0.155	0.961	0.674	0.711
	ARI ^b^	rho	0.135	0.067	−0.003	−0.284
		*p*-value	0.618	0.806	0.991	0.286

*p* < 0.05; ^a^ Pearson’s correlation; ^b^ Spearman’s correlation.

## Data Availability

The original contributions presented in this study are included in the article. Further inquiries can be directed to the corresponding authors.

## Supplementary Materials

The following supporting information can be downloaded at: https://www.mdpi.com/article/10.3390/jfb16010007/s1, Table S1: Descriptive data of the length and width of enamel microcracks before bonding and after debonding.

## Abbreviations

OCT: optical coherence tomography; EMCs: enamel microcracks; 2D: two-dimensional; 3D: three-dimensional; DEJ: dentin–enamel junction; SEM: scanning electron microscopy; QLF: quantitative light-induced fluorescence; µCT: micro-computed tomography; SD-OCT: spectral-domain optical coherence tomography; FWHM: full width at half-maximum; ARI: adhesive remnants index; SBS: shear bond strength; ROI: region of interest.
