# Supplementary material for: Three-Dimensional Assessment of Dental Enamel Microcrack Progression After Orthodontic Bracket Debonding Using Optical Coherence Tomography
Source: J Funct Biomater. 2024 Dec 30;16(1):7. doi: 10.3390/jfb16010007 (PMC11766427; doi:10.3390/jfb16010007)
Supplement: Supplementary file 1 [file jfb-16-00007-s001.zip › jfb-3289838-supplementary.pdf]

Table S1. Descriptive data of the length and width of enamel microcracks before bonding and after debonding.

Group	Sample ID	EMCs length		EMCs width	
		Before	After	before	After
Ceramic	Sample 1	4.86	5.53	117.00	442.00
	Sample 3	3.34	5.10	143.00	533.00
	Sample 5	1.75	2.95	312.00	390.00
	Sample 6	4.70	5.70	156.00	351.00
	Sample 8	8.37	8.37	117.00	338.00
	Sample 10	4.00	4.86	182.00	299.00
	Sample 12	4.15	5.74	156.00	221.00
	Sample 14	2.49	3.20	195.00	221.00
	Sample 16	5.17	7.80	130.00	416.00
Metal	Sample 2	6.92	6.92	143.00	416.00
	Sample 4	7.08	7.08	156.00	260.00
	Sample 7	3.54	4.14	260.00	624.00
	Sample 9	4.06	4.60	208.00	312.00
	Sample 11	4.10	5.75	195.00	299.00
	Sample 13	2.80	3.82	104.00	260.00
	Sample 15	3.28	3.70	234.00	377.00
